# The Relationship Between a Baby's Age and Sleepiness in a Sample of Mothers

**DOI:** 10.3389/fpsyg.2021.694884

**Published:** 2021-07-02

**Authors:** Mar Sánchez-García, María José Cantero, Eva Carvajal-Roca

**Affiliations:** ^1^Departamento de Psicología Evolutiva y de la Educación, Universitat de València, Valencia, Spain; ^2^Pediatría, Hospital de la Salud, Valencia, Spain

**Keywords:** motherhood, fatigue, sleepiness, baby, age, developmental psychology

## Abstract

One question of great practical importance for the parents, and especially the mother, after the birth of a baby, refers to how long the time during which they have to go with less and more fragmented sleep actually lasts. Most of the studies only explore this issue up to 6 months of the newborn's life, and less is known about the sleep problems the mothers may have after this initial period. The objective of this study is to examine the relationship between the sleep disruption and daytime sleepiness of mothers with infants until 2 years old compared to a group of women currently not at care of babies. To this end, a sample of 113 women, 67 currently bringing up a baby of under 2 years old, and the remainder without a baby at their care under 6 years old, reported sleep duration, sleep interruptions, sleep quality, and responded to questionnaires of sleep quality and daytime sleepiness. The relationship between the age of the children and the comparison between the groups was used to highlight the sleep problems of the mothers taking care of the infant. The results showed that there was a positive relationship between the age of the infant and the duration of the sleep of the mothers and that the duration of sleep for them was similar to those of the women in the control group about 6 months after the infant was born. However, fragmentation of sleep, daytime sleepiness, and sleep problems were still higher than in the control group for mothers with children between 6 and 12 months old.

## 1. Introduction

The birth of a child marks the beginning of a new phase in the lives of both parents, particularly for the mother. Throughout the first months in the life of a baby, the mothers undergo numerous changes, psychological, physiological and behavioral, and although these are partly understood, there are aspects to them that deserve to be studied in depth.

One question of great practical importance refers to how long the time during which mothers and fathers have to go with less and more fragmented sleep actually lasts. “Will this go on for 1 month, two, or even longer?” is something they often ask themselves. The reason for this is that, if an answer was available, parents could make crucial decisions, such as whether to call in extra help, return to work, carry on studying etc. What is more, when any positive expectations regarding maternity (among which is usually found the hope that the children “will sleep well” from an early age) are left unfulfilled, this leads to feelings of frustration. Nevertheless, given that this information usually comes from informal sources, such as friends, family or other parents of newborn children, it may often be positively distorted and give rise to expectations that are not realistic. This is why it may be of great value to determine the length of this time objectively so as to be able to offer help to those who are going through this very situation enabling them to face up to it as best they can.

One estimation of the time that it may take the parents to return to their previous sleep patterns has usually been based on the postpartum, the duration of which is usually established as lasting for the first 6 months of a baby's life. It starts with the birth and the expulsion of the placenta-responsible for the secretion of many hormones that may alter the normal rhythms-and continues during lactation and until the child's sleep follows some predictable sleep-wake cycle patterns (Lee, 1998). Research into maternal sleep deprivation is usually centered around this time. For example, in an exhaustive review, Hunter et al. (2009) only mention studies in which the newborns are under 3 or 6 months old (Quillin, 1997; Matsumoto et al., 2003; Signal et al., 2007), and most of the recent studies published on this topic still focus on this period (Creti et al., 2013, 2017; Tran et al., 2015; Kenny et al., 2020; Cattarius and Schlarb, 2021; Da Costa et al., 2021). However, two recent studies have broken this trend and have provided evidence regarding parent's sleepiness when the newborns are over 6 months. So, Sivertsen et al. (2015) interviewed women 2 years after postpartum and found that “a large proportion still fulfilled the diagnostic criteria for DSM-IV insomnia,” and Richter et al. (2019) examined changes in mothers' and fathers' sleep before pregnancy and the postpartum period of up to 6 years after birth and observed that, for the first childbirth, they had not yet fully recovered sleep satisfaction and hours of sleep at the end of the study. However, these studies use interviews set at specific points so that they do not provide fine-grained information about how the sleep indicators change in relation to the age of infants.

Although it is true that the internal factors determining the sleep-wake cycle in children do develop between 3 and 6 months of age and that mothers tend to sleep more as the child develops, focusing on this period of postpartum overlooks the fact that there other external factors that affect the mothers well-being: the style of child-rearing, for instance, whether the parents and child co-sleep, the type of lactation, the general organization of the household, the work outside the home, etc., all of these factors may continue to alter a mother's normal sleep rhythms. Moreover, it should not be forgotten that even after the children have managed to achieve an acceptable sleep rhythm, the mothers may still find it difficult to get enough sleep due to psychological problems that may arise associated with the pregnancy and the perinatal period (Fallon et al., 2016). That said, since there is evidence that sleeping habits —including the time people normally go to bed, the time they get up, and the time that passes between these moments— vary according to country and gender (Walch et al., 2016), it is of interest to evaluate whether the abovementioned results are reproduced in Spanish samples.

There are several milestones in the development of children after the 6 months of birth that may contribute to reducing the burden of the parents associated with caring for them. At 10-month postpartum the child's language begins to develop; at 14 months postpartum, the child is able to walk, to explore the environment, and attachment behavior is at its peak (Prenoveau et al., 2017). At 24 months old the children are more independent, have sufficient language skills to communicate with others which helps to manage their negative emotions, and can focus their attention away from stressful stimuli in order to manage their distress as well as to soothe themselves (Spinrad et al., 2004; Dennis, 2006). Thus, it seems of interest to extend the research of the sleep disturbances of mothers until at least the children are up to 24 months old.

There are several types of sleep disturbances that can affect the mothers such as sleep reduction, fragmentation of sleep, daytime sleepiness, etc. One well-identified problem is that of sleep deprivation, which is something common to mothers throughout the first months in the life of their baby (Hunter et al., 2009) and which is associated with the infant's nutrition, care, and sleep rhythm: since the sleep rhythms of the newborn are still not well-established and their need for nourishment, affection, cleanliness, and activity is not yet synchronized with the rhythm of the parents, both the mother and father are often sleep-deprived, in terms of both quantity and quality. This, in turn, may lead to there being many adverse effects on, among other things, their psychological and physical health, and their social relations (Moline et al., 2003; Sharma and Mazmanian, 2003). For example, Okun et al. (2018) found that symptoms of depression and anxiety in a group of 116 women were related to having poor sleep quality. Tham et al. (2016) found that poor subjective sleep quality during pregnancy was associated with borderline high postnatal depressive symptoms highlighting that the origin of the problem might be caused by disturbances that happen before birth delivery. Understanding and mitigating the impact of this sleep disruption is important for the health of the mother, as a large amount of wake after sleep onset and low sleep efficiency are predictive of postpartum fatigue severity and mood in general (Posmontier, 2008; Bei et al., 2010). New mothers usually do not expect the sleep disruption that they experience (Kennedy et al., 2007).

Disruption of sleep at night may cause daytime sleepiness, which may affect the daily activities of the mothers or turn into low productivity on the job and on accident rates, both occupational and non-occupational (Lee, 1998). At the beginning of the postpartum, mothers have less nighttime sleep and they spend a greater time awake following sleep onset (Gay et al., 2004), but it is sleep disruption rather than total sleep obtained that is more influential in daytime sleepiness (Insana and Montgomery-Downs, 2010). For instance, although Montgomery-Downs et al. (2010b) found that the quantity of sleep obtained by new mothers from postpartum weeks 2–16 was relatively consistent (7.2 h), the sleep quality improved over the same period due mainly to a reduction in sleep fragmentation and increase in sleep efficiency. However, this reduction in sleep fragmentation did not happen in the study of Filtness et al. (2014), as they did not observe significant differences in frequency of awakenings during nocturnal sleep between the weeks 6, 12, and 18, with averages of 1.9, 1.65, and 1.7, although they found differences in other indicators.

The objective of this study is to ascertain whether the quantity and quality of sleep in a sample of mothers of newborns in Spain during the first 2 years of the baby's life differ from that of women of similar characteristics, but who are not raising a child, and to analyze whether any difference is limited to the first semester or carries on beyond this period.

## 2. Materials and Methods

The design of this study corresponds to the description in Shadish et al. (2002) of a quasi-experimental post-test only study. Two groups (one treatment group and one control group) took part in the study, which was retrospective and questionnaire-based. The study consisted of obtaining answers from two groups of women to the questionnaires described in the measures section. Prior to the procedure, written consent was obtained from all of the participants for the aggregate use of the results for research purposes. This study was conducted following the guidelines set out by our institution.

To this end, a convenience sample of 113 women, 67 currently bringing up a baby of under 2 years old, and the remainder without a baby under 6 years old in their care, were interviewed as to a series of relevant variables related to the quantity and quality of sleep they usually enjoyed. The answers were assessed in order to evaluate the hypothesis that both the quality and quantity of sleep enjoyed by women with a baby in their care were reduced when compared to women who did not have a baby in their care during not only the first semester of the newborn's life but also the subsequent ones.

The participants were not furnished with any additional information on the study's objectives before their answers were registered. Once they had finished, the objective of the study was succinctly explained to them and they were compensated for the effort they had made in participating (30 €).

### 2.1. Participants

Women with babies in their care and willing to participate in a study into sleepiness were contacted while in the waiting room of a pediatrician, one of the co-authors of this article, located in a hospital in the city of Valencia. They were required to be between 25 and 50 years of age and to have no serious health problems. Additionally, their children had to be between 1 and 24 months old.

The women who had no babies in their care were firstly contacted in the pediatrician's waiting room but also through friends, relatives and workmates of the women who had already taken part in the study. The requirements for these women were the same as for the first group, except that they should not have children or that their children had to be at least 6 years old.

The total number of participants in the study was 113, of whom 67 met the requirements necessary to be classified as having children in their care. From now on, this sample will be referred to as “With infants” (for the women with babies in their care). The remaining 46 met the requirements needed to be classified as having no babies in their care and we shall refer to them as “Control” (for the women not currently with babies in their care).

### 2.2. Measures

Two types of variables were measured in this study: standardized questionnaires related to sleepiness and direct questions concerning night sleep. In addition, the demographic and behavioral variables of the participants were evaluated through *ad-hoc* questions.

#### 2.2.1. Sleepiness Scales

Three scales were used to evaluate the mothers' sleepiness.

- General Sleep Disturbance Scale (GSDS): This scale (Lee, 1992; Shahid et al., 2012) was designed to evaluate the incidence and nature of sleep alterations in working women. The GSDS is a self-report, paper- and-pencil measure requiring 5–10 min for completion. The GSDS queries respondents regarding the frequency with which they've experienced certain sleep difficulties within the previous week. Respondents use an eight-point, Likert-type scale ranging from 0 (meaning “never”) to 7 (“every day”) to respond to each item. The GSDS is a 21-item scale initially designed to evaluate the incidence and nature of sleep disturbances in employed women. Questions pertain to a variety of general sleep issues, including problems initiating sleep, waking up during sleep, waking too early from sleep, quality of sleep, quantity of sleep, fatigue and alertness at work, and the use of substances to induce sleep. Researchers have suggested that individuals with an average score of three (averaged by number of items) on the GSDS should be considered at risk for sleep disturbance (following guidelines set in the Diagnostic and Statistical Manual of Mental Disorders). A psychometric evaluation of the scale carried out by Lee (1992) found an internal consistency of 0.88 for the whole scale. This scale was used in Sánchez-García (2017).
- Epworth Sleepiness Scale (ESS): This scale (Johns and others, 1991) was designed to evaluate daytime sleepiness by asking the respondents to evaluate the probability of falling asleep in eight different situations. It uses a 0–3 scale. Scores range from 0 to 24, with higher scores indicating a higher propensity for sleepiness and a score of >10 indicating excessive daytime sleepiness (Johns and Hocking, 1997). Each situation represents a moment of relative inactivity, from sitting down reading to sitting in a stationary car at a traffic light. The scale was validated using an adult population of between 18 and 78 years old. As far as its reliability and validity are concerned, it exhibits high internal consistency and good test-retest reliability Test-Retest Reliability of the Epworth Sleepiness Scale in a Sleep Clinic Population (Lee et al., 2018); it also correlates positively with the probability of falling asleep at the wheel (Maycock, 1997). The ESS is significantly correlated with sleep latency as measured by objective measures such as the multiple sleep latency test (MSLT) and overnight polysomnography (PSG), and it can detect changes following continuous positive airway pressure (CPAP) treatment (Johns and others, 1991). It remains one of the most widely used measures of habitual daytime sleepiness (Kaplan and Gasperetti, 2020).
- Karolinska Sleepiness Scale (KSS): The Karolinska Sleepiness Scale(KSS) is a single-item self-report measure of situational “state” sleepiness (Åkerstedt and Gillberg, 1990). This scale measures the subjective level of sleepiness state in the last 10 min. The KSS measures situational sleepiness and, therefore, is sensitive to momentary fluctuations that occur in short time periods. Individuals rate their current level of alertness on a 9-point ordinal scale (1 = “extremely alert,” 5 = “neither alert nor sleepy,” 9 = “extremely sleepy—fighting sleep”). The KSS is strongly correlated with time of day, and scores increase as the period of wakefulness extends (Kecklund and Akerstedt, 1993). The KSS is significantly correlated with electroencephalographic (EEG) and the psychomotor vigilance task (PVT), indicating that it is a valid measure of sleepiness (Kaida et al., 2006). This scale was used in studies into shift-workers and drivers and is useful to evaluate any changes in the response to environmental influences, circadian rhythm and the effects of drugs. In a validation study, Kaida et al. (2006) found a close correlation between electroencephalographic measurements and behavioral variables. KSS is indicated as a measure of momentary sleepiness (Kaplan and Gasperetti, 2020).

There are many scales for measuring sleepiness (over a 100 according to Shahid et al., 2012) and, as a consequence, choosing one over the others can be difficult—for a recent review of the pros and cons of 24 of these measures see Kaplan and Gasperetti (2020)—. The three scales mentioned before were chosen because they measured different aspects of sleep disruption. The GSDS asks mainly for nighttime sleep problems in the past week such as awakenings, use of alcohol or other substances for sleeping, and sleep satisfaction (Lee et al., 1991). The ESS instead is indicated for measuring habitual daytime sleepiness or average sleep propensity (Johns, 2008). Finally, the KSS asks for momentary sleepiness at the time of responding (Kaplan and Gasperetti, 2020). As the participants filled the questionnaires during the mornings, approximately between 10 a.m. and 1 p.m. the scores in the KSS can be taken as an indicator of sleepiness in the first part of the day.

#### 2.2.2. Evaluation of Night Sleep

We mentioned in the introduction that disruption of sleep in mothers may happen in different ways: reduction of time and fragmentation of sleep being the most common. Fragmentation is the result of awakenings in response to noises made by the baby, that may involve getting off the bed in some cases. Also, mothers can be too alert during the night for deep sleep and experience a low quality in their sleep as a consequence. Nighttime number of hours of sleep is also important but it is common among mothers to recover some of the sleep lost with naps taken in the daytime. Researchers that have explored these aspects of maternal sleep have often used diaries in which mothers recorded the events related to sleep (Insana and Montgomery-Downs, 2010; Filtness et al., 2014). However, as our study was based on an interview we simply asked questions directly to the participants. The questions in our case were:

- How many times do you wake up during the night?
- How many times do you get up during the night?
- How would you rate the quality of your sleep? (Ratings from 1 = very bad, 2 = bad, 3 = normal, 4 = good, 5 very good)
- In total, how many hours do you sleep a night?
- The number of hours you sleep during the day (naps).
- The total number of hours of sleep: Unlike the previous questions, this was not asked directly but was obtained by adding up the answers to the previous two questions.

### 2.3. Data Analysis

For data analysis purposes, the scores from the scales were added up after inverting the negative items. The data were analyzed graphically (scatter and box plots), *t*-tests, and analysis of variance, using the correction for non-homogeneity of variance (Welch or Games-Howell). Post hoc comparisons were adjusted using the Holm's procedure. Significance was always evaluated at *p* < 0.5. All of the calculations were done and the graphs plotted using R (Team, 2020). There was a very small number of missing values, but given that the analyses were exclusively univariate, the impact was very limited.

**Figure 1:**
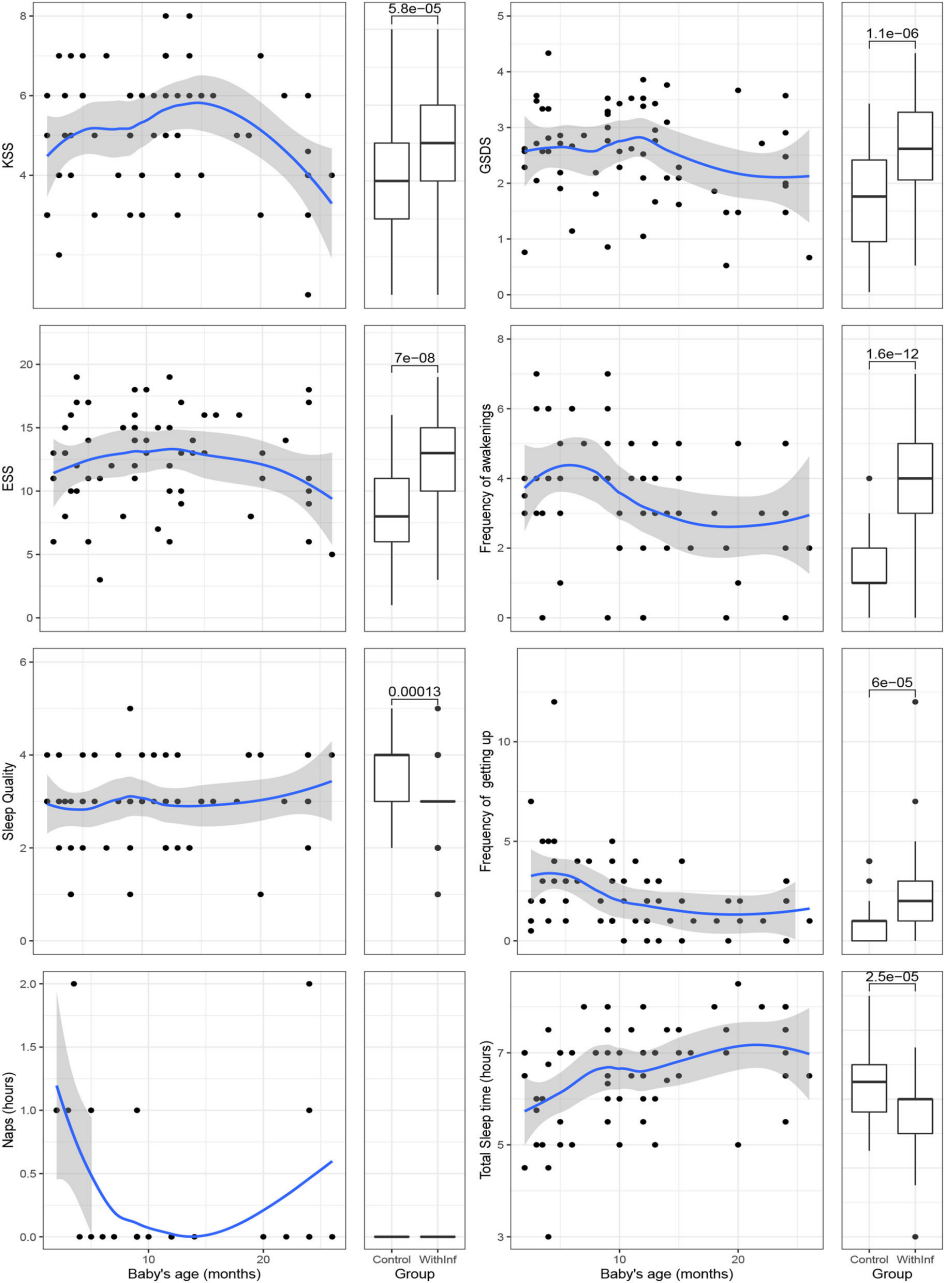
Scatter plots showing relationship between sleepiness variables in mothers with babies of under 24 months old and the normal values of women without babies and box plots showing the same comparison.

The relationship between the age of the baby and the indicators of sleepiness is displayed graphically in Figure 1. This figure has a couple of plots per indicator, with a total of eight indicators corresponding to the three sleepiness scales plus the answers to the questions evaluating night sleep. The first plot of each couple is a scatterplot in which the age of the baby is set in the horizontal axis and the indicator is set in the vertical axis. As the relationship between the age of the baby and the indicators was found to be non-linear in many cases, we overimposed a non-parametric loess curve in all the plots to better visualize the relationship between the variables (Cleveland and Devlin, 1988). The second plot of the couple is a boxplot for comparing the two groups of women in our study: the control group (mothers without infants under 24 months old at the time of the study) and mothers with infants. Each boxplot shows overimposed the result of a *t*-test comparing the means of the groups in each indicator. As the scatterplots and the boxplots share the same vertical axis, it is possible to assess when the values of the scatterplot are over or under the median values of the indicators for the two groups of mothers.

**Figure 2:**
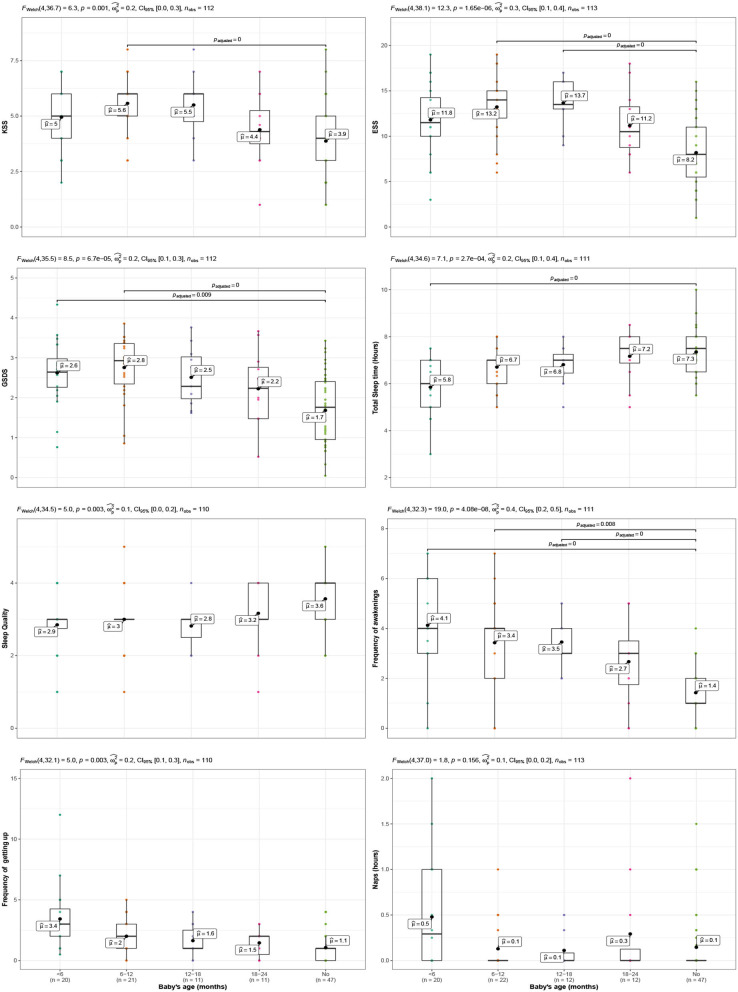
Comparison of sleepiness variables in two cases: that of mothers of newborn babies, whose ages are split into four groups (groups <6 months old, 6–12 months old, 12–18 months old, 18–24 months old) and that of women who have not been mothers recently (No group). Each variable is shown in a panel and each of the panels includes information on the result of an ANOVA test, eta squared, its confidence interval, the sample size, and inter-group comparisons -only shown if significant- assuming unequal variances (Games-Howell) and adjusting for the number of comparisons using the Holm's method.

Additionally, the mothers were grouped according to if their babies were <6 months old, between 6 and 12, 12 and 18, and 18 and 24 months old. ANOVA tests for each indicator among these four groups plus the women in the group control are shown in Figure 2. This figure has a plot per indicator, with five boxplots in each of the plots. Means are displayed with a black dot overimposed on the boxplot. Results of ANOVA tests are shown on the top of each plot. *Post-hoc* comparisons are shown overimposed on the plots with lines indicating pairs of groups whose mean were significantly different between them.

## 3. Results

There are four parts in this section: Sample description in terms of demographic variables, description of sleepiness variables, comparison between groups of women and relationship with the age of the baby, and comparison of women according the baby's ages groups.

### 3.1. Sample Description

Table 1 shows a comparison between the samples of the two groups of women participating in the study. As can be seen, the age of the two sample groups was similar. In the group without babies, the percentage of women who were married or in an equivalent relationship was slightly lower than in the group with babies, which is what could be expected given that women with infants are more often in a relationship than women without infants. It would be interesting to check the sleepiness of mothers without significant others supporting them but we only had 4 in our study so this was not possible in our case. The percentage of unemployed women (and, therefore, the opposite case, of women who were in a job when the study was carried out) was similar. Lastly, both samples of women were generally free from health problems except for one case, but after an in-depth analysis of this case we included her as the problem did not affect her day-to-day activity.

**Table 1:** Description of the samples of women taking part in the study who did not have a baby of under 24 months old in their care when the study was carried out (Control) and of those who did (With Infants).

**Descriptive variables**	**Total N**		**Control**	**With infant**	**Total**	**Stat**	***p***
Age	111	Mean (SD)	36.9 (7.9)	34.6 (5.1)	35.5 (6.5)	1.8	0.061
Age of first child	87	Mean (SD)	12.4 (7.7)	12.6 (10.0)	12.6 (9.5)	−0.1	0.909
Married or equivalent relationship	111	Not	10 (22.2)	4 (6.1)	14 (12.6)		0.026
		Yes	35 (77.8)	62 (93.9)	97 (87.4)		
Unemployed?	113	Yes	9 (19.6)	17 (25.4)	26 (23.0)		0.622
		Not	37 (80.4)	50 (74.6)	87 (77.0)		
Currently employed or maternity leave	88	Full time	19 (51.4)	17 (33.3)	36 (40.9)	13.2	0.001
		Part time	18 (48.6)	19 (37.3)	37 (42.0)		
		Maternity leave	0 (0.0)	15 (29.4)	15 (17.0)		
Health problems	112	Yes	0 (0.0)	1 (1.5)	1 (0.9)		1.000
		Not	45 (100.0)	66 (98.5)	111 (99.1)		
Total N (%)			46 (40.7)	67 (59.3)	113		

*p value corresponds to a mean difference t-test or a chi-squared test*.

As this was a pilot study and the sample size was not large, we could not test the relationship between some of the demographic variables and the sleepiness of the mothers with infants.

### 3.2. Description of Sleepiness Variables

Table 2 shows the descriptives for the sample used in this study.

- GSDS: The mean is 2.2, below the threshold of three that is considered as at risk of sleep disturbance. The standard deviation is 0.9. The minimum value was one and the maximum is 4.3, and 50% of the scores lying between 1.5 and 2.9. This variable was very close to normal as its asymmetry is close to 0 (–0.2).
- ESS: The mean is 10.7, which is slightly over the threshold of excessive sleepiness (10). The standard deviation is 4.2. The minimum value is 1 and the maximum 19, and 50% of the scores lying between 8 and 14. This variable is very close to normal as its asymmetry is close to 0 (*Skew* = −0.1).
- KSS: The mean is 4.6 which is between the “Rather alert” and “Neither alert nor sleepy” categories of this scale. The minimum value is one and the maximum is eight. This variable is very close to normal as its asymmetry is close to 0 (*Skew* = −0.1).
- Quality of sleep: The mean is 3.2 which is near the “Normal” category. The minimum is one and the maximum is five. This variable was is close to normal as its asymmetry is close to 0 (*Skew* = −0.5).
- Frequency of awakenings: The average frequency of awakenings was 2.6, with a minimum of zero and a maximum of 7. This variable is very close to normal as its asymmetry is close to 0 (*Skew* = 0.4).
- Frequency of getting up during the night: The mean was 1.8, with a minimum of zero and a maximum of 12. This variable shows positive asymmetry (*Skew* = 2.3) due to the outlier.
- Total hours of sleep by night: The mean was 6.7 h, with a minimum of 3 and a maximum of 9. This variable is moderately asymmetric (*Skew* = −0.8).
- Minutes of naps per day: The mean is 12, with a minimum of 0 and a maximum of 120 min. This variable shows positive asymmetry (*Skew* = 2.3).
- Total hours of sleep: This variable was computed summing the two previous variables. The mean is 6.9. The minimum is 6.3 and the maximum is 10. This variable is very close to normal as its asymmetry is close to 0 (*Skew* = −0.5).

**Table 2:** Descriptive statistics for the sleepiness variables.

**Variable**	**Mean**	***SD***	**Min**	**Q1**	**Med**	**Q3**	**Max**	**Asimmetry**	**valid n**	**valid pct**
**Scales**										
GSDS: Do you experience these problems when getting to sleep?	2.2	0.9	0	1.5	2.3	2.9	4.3	–0.2	112	99.1
ESS: What is the likelihood of you falling asleep in situation x?	10.7	4.2	1	8.0	11.0	14.0	19.0	–0.1	113	100.0
KSS: Level that reflects your state in the last 10 min.	4.6	1.6	1	3.0	5.0	6.0	8.0	–0.1	112	99.1
**Questions about nigh sleep**										
How would you rate the quality of your sleep?	3.2	0.8	1	3.0	3.0	4.0	5.0	–0.5	110	97.3
How many times do you wake up during the night?	2.6	1.8	0	1.0	2.0	4.0	7.0	0.4	111	98.2
How many times do you get up during the night?	1.8	1.7	0	1.0	1.0	3.0	12.0	2.3	110	97.3
In total, how many hours do you sleep a night?	6.7	1.1	3	6.0	7.0	7.5	9.0	–0.8	111	98.2
How long are your naps during the day (in minutes).	12.8	25.3	0	0.0	0.0	20.0	120.0	2.3	113	100.0
Total hours of sleep	6.9	1.1	3	6.3	7.0	7.5	10.0	–0.5	111	98.2

As a whole, the data did not show special characteristics apart from one outliers in number of awakenings (7) and other in times getting up (12). Interestingly, some women got up from bed more times than they woke up by night, which can be interpreted as that despite of lying in bed they remained alert without actually falling slept.

### 3.3. Comparison Between Groups of Women and Relationship With the Age of the Baby

Figure 1 shows the comparison of the women in the group control with the mothers with infant babies (under 24 months old). We will comment on the figures from left to right, top to bottom:

- KSS: The mothers with infant babies with ages under 6 months old had scores near 5. Mothers with babies between 6 and 12 months old had higher scores than those with younger babies and older babies. Mothers with babies older than 12 months old had scores close to the group of mothers with infant babies under 6 months old and the relationship decreases for mothers with babies over 18 months old. The scores for the mothers in the KSS are over the median of the women in the control group except for mothers with children over 18 months old. The global differences between the two groups of women were significant [Δ*M* = −1.26, 95% CI [−1.85, −0.67], *t*_(90.92)_ = −4.22, *p* < 0.001] with women in the control group having lower scores than mothers attending infant babies.
- GSDS: The scatterplot for the GSDS measure shows values between 2 and 3 for mothers with babies under 1 year old, which is below the score of 3, regarded as the threshold for problems of sleep, although the 95% interval of confidence band overimposed on the loess line includes this value during the first year. Then, there is a decrease in scores for mothers with babies over 12 months old but in average they are still higher than the scores of women without infants. The global differences between the two group of women were significant [Δ*M* = −0.84, 95% CI [−1.16, −0.52], *t*_(97.37)_ = −5.21, *p* < 0.001] with women in the control group having lower scores than mothers attending infant babies.
- ESS: The scatterplot for the Epsworth questionnaire measure shows that the mothers with children have scores generally higher than the threshold of 10, which is regarded as the cut-off for sleep problems. The loess curve bends down for mothers with babies over 18 months old, remaining stable for children under this age. The global differences between the two group of women were significant [Δ*M* = −4.13, 95% CI [−5.53, −2.73], *t*_(96.02)_ = −5.84, *p* < 0.001] with women in the control group having lower scores than mothers attending infant babies.
- Frequency of awakenings: Mothers with infant children under 10 months old reported waking up about four times per night, but those with children older than 10 months had only approximately three awakenings per night. The frequency of awakenings for mothers with 24 months old babies was higher than for women without infants. The global differences between the two group of women were significant [Δ*M* = −2.07, 95% CI [−2.58, −1.56], *t*_(103.46)_ = −8.03, *p* < 0.001] with women in the control group having 1.41 awakenings in average vs. 3.48 awakenings of mothers.
- Sleep quality: The relationship between the sleep quality of the mothers and the age of their infants is close to zero as the loess line in the plot is almost horizontal. The global differences between the two group of women were significant [Δ*M* = 0.59, 95% CI [0.29, 0.88], *t*_(104.95)_ = 3.98, *p* < 0.001].
- Frequency of getting up: Mothers get up by night more times when their infants are under 5 months old. One case stands out because one mother reported getting up 12 times per night. Mothers with children over 5 months old children drop the frequency in which they get up. The global differences between the two group of women were significant [Δ*M* = −1.21, 95% CI [−1.78, −0.64], *t*_(100.64)_ = −4.19), *p* < 0.001], with women in the control group having an average frequency of 1.07 in the control group vs. a frequency of 2.27 in the group of mothers with infants.
- Naps: The naps that mothers took varied considerably among them. Two of them reported 2 h of nap and five reported 1 h but the majority indicated zero o close to zero nap time. No discernible relationship between the age of the infant and the nap duration was observed. Also, the differences between the women with and without infants were not significant [Δ*M* = −6.53, 95% CI [−15.54, 2.47], *t*_(110.72)_ = −1.44, *p* = 0.153].
- The total sleep time: The total sleep time was calculated by summing the hours slept by night plus the naps. The relationship between the age of the babies and the total slept time of their mothers is positive and only bends down slightly once the children are 20 months old or over. However, at this point, the mothers slept about the same as the women in the control group. The differences between the two groups were significant [Δ*M* = 0.79, 95% CI [0.42, 1.16], *t*_(107.25)_ = 4.25, *p* < 0.001] with women in the control group having an average frequency of 7.34 in the control group vs. a frequency of 6.55 in the group of mothers with infants.

### 3.4. Comparison Between Babies' Age Groups

The results until here suggest that the mothers may experience sleepiness-related problems that last beyond 6 months, which is what is considered to be the moment when the babies should have regularized their sleeping habits, and consequently their mothers could return to previous sleep habits. In order to verify this hypothesis more accurately, the mothers with infants were split into four groups according to the age of their babies: from 0 to 6, 6 to 12, 12 to 18, and 18 to 24 months. The group of mothers without babies (Control group) was added to these four groups and comparisons were carried out between these five groups through ANOVAs and a posteriori comparison tests (assuming unequal variances and adjusting for the number of comparisons using the Holms method). The results are summarized in the eight panels of Figure 2, in which the upper part of each panel shows the result of the analysis of variance, the size of the effect, its confidence interval, and the number of cases used each time.

- KSS: The results of the ANOVA were significant [(*F*_(4, 36.73)_ = 6.34), *p* = 0.001] with eta-squared equal to 0.2. The group of mothers with infants between 6 and 12 months old was different from the control group but any other group showed differences.
- ESS: The results of the ANOVA were significant [*F*_(4, 38.10)_ = 12.28, *p* < 0.001] with eta-squared equal to 0.3. The group of mothers with infants between 6 and 12 months old, and between 12 and 18 months old showed differences with the control group.
- GSDS: The results of the ANOVA were significant [*F*_(4, 35.46)_ = 8.46), *p* < 0.001] with eta-squared equal to 0.2. The group of mothers with infants between under 6 months old, and between 6 and 12 months old showed differences with the control group.
- Total sleep time: The results of the ANOVA were significant [*F*_(4, 34.62)_ = 7.12, *p* < 0.001] with eta-squared equal to 0.2. The *post hoc* comparison showed differences between the group of mothers with infants under 6 months old and the control group.
- Sleep quality: The results of the ANOVA were significant [*F*_(4, 34.49)_ = 5.01, *p* = 0.003] with eta-squared equal to 0.1 but its interval of confidence included 0 *CI* = [0, 0.2]. Also, the *post hoc* comparison did not show differences between any of the groups.
- Frequency of awakenings: The results of the ANOVA were significant [*F*_(4, 32.33)_ = 18.97, *p* < 0.001] and the eta-squared was equal to 0.4. All the groups of mothers showed significant differences from the control group except the one with babies between 18 and 24 months old.
- Frequency of getting up: The results of the ANOVA were significant [*F*_(4, 32.15)_ = 4.99, *p* = 0.003] with eta-squared equal to 0.2. However, the *post hoc* comparison did not show differences between any of the groups.
- Naps: The results of the ANOVA were not significant [*F*_(4, 37.01)_ = 1.77, *p* = 0.156].

## 4. Discussion

The results have shown that mothers with infants have their sleep disrupted in comparison with a group of normal women. So, the mothers had higher scores with respect to nighttime sleepiness problems (GSDS), daytime sleepiness (Epworth), and current sleepiness (KSS). Also, they woke up more times per night, rated their sleep quality lower than the control group, got up more times, and slept fewer hours. Only the time employed in naps did not differ between the mothers and the control group.

The relationship between the sleepiness variables and the age of the infant was non-linear for many of the indicators. So, the nighttime sleep problems (GSDS), the daytime sleepiness (ESS), and the instant sleepiness (KSS) were at their highest point when the infants were about 10 months old. The frequency of awakenings was at its highest point when the children were <6 months, although it remained significantly different from the control group for mothers with children between 6 and 18 months old. The sleep quality ratings however did not show any relationship with the age of the infant, while the total hours of sleep increased almost linearly.

The results are consistent with previous research in several aspects. So, the reduction in sleep during the first months after birth is well established (Gay et al., 2004) and we have found it also in this study. Reduction of sleep after the first 6 months is less studied but (Sivertsen et al., 2015) found that the sleep duration and the time in bed of the mothers at 2 years postpartum were higher than at 8 weeks. Richter et al. (2019) also found that the reduction of sleep was significant after 1 year of birth respect to 1 year before pregnancy and only recovered partially, but not at levels before birth, after the second year. This result is not consistent with ours, as in our case the sleep duration of the mothers with 1 years old children was similar to the women not taking care of infant children but the different methodology (longitudinal study vs. cross-sectional) might lead mothers to provide the information in a different way. A sound methodological design would involve following two groups of women (with and without babies) during the same period of time so that comparisons within and between groups could be carried out.

We did not find a relationship between the rating of the quality of sleep and the age of the infant. Sivertsen et al. (2015) also did not find differences between the week 8 and the year 2 postpartum in two similar indicators (non-restorative sleep, and sleep dissatisfaction), but they found differences with pre-pregnancy levels. Richter et al. (2019) found that the first year was characterized by low levels of sleep satisfaction in comparison with years 2–6 but differences were not significant between 1 and year 2. So, in summary, it looks like the mothers' perception of an improvement in quality of sleep in mothers does not come probably until after the second year postpartum. This finding should be confirmed with more studies that followed mothers for more than the 2 years limit used in this study.

That the subjective perception of quality of the sleep of mothers does not improve as quickly as the duration of the sleep has been connected with sleep fragmentation (Insana and Montgomery-Downs, 2010; Montgomery-Downs et al., 2010a). In our case, mothers with infant children had more awakenings than women without children with an average of 4 vs. 1. The peak of this problem occurred during the first 6 months of the postpartum period, decreasing afterwards. However, the average of awakenings was still well above of the women without infant children for all the groups of mothers with children under 18 months old. There were also significant differences in the frequency that the mothers got up during the night but no specific age of the children was identified as the cause of this difference.

One important consequence of sleep disruptions is the daytime sleepiness that mothers may experience. This sleepiness may reduce their performance in many tasks and may constitute a safety hazard in the job (Lee, 1992). Our results point out that the KSS, which is an indicator of the instant sleepiness, had its highest value when their children were between 6 and 12 months old. The Epworth daytime sleepiness was also significantly different for the mothers with children between 6 and 12 months and 12–18.

All in all, our study puts into question the assumption that any sleepiness-related problems a mother may have will be resolved by the end of the first semester of a baby's life. So, our results signal that the mothers with children of 12 months old are the ones with more sleep problems, interruptions, and daytime sleepiness despite that their sleep time is not at its worst.

There are several limitations to this study that we shall now comment on. Firstly, both the size of the sample and the method of gathering data mean that generalization to a the reference population cannot be ensured; so, this study should be considered as a pilot study which would mainly be of interest to lay the foundations for a wider-ranging study to evaluate the problems related with sleep-deprivation suffered by mothers of newborns in Spain. Secondly, the variables analyzed herein essentially refer to sleepiness and, although it is known that this is closely related to fatigue, either of which may occasionally be cause or consequence of the other, they should not be mixed up as there is at least one very important difference: fatigue may become chronic and not even rest may help a person to recover from it whereas sleepiness can be alleviated more quickly (Shen et al., 2006). Another limitation is related to the use of subjective measures, which could be substituted for more objective measures using actigraphy that recorded the sleep cycles.

Lastly, this study does not include a bigger number of variables which would help to understand why problems of sleepiness persist beyond the postpartum even though the initial reason, the baby's lack of maturity, should no longer represent a problem. Our recommendation, therefore, is that future research into sleepiness-related problems in mothers should enlarge the size of the sample, improve the sampling method and cover a broader spectrum of variables. Ideally, this research should be longitudinal, include a control group, and follow the evolution of the mothers both before and during the birth and throughout the child-rearing phase for as long as possible.

## Data Availability Statement

The raw data supporting the conclusions of this article will be made available by the authors, without undue reservation.

## Ethics Statement

Ethical review and approval was not required for the study on human participants in accordance with the local legislation and institutional requirements. The participants provided their written informed consent to participate in this study.

## Author Contributions

All authors listed have made a substantial, direct and intellectual contribution to the work, and approved it for publication.

## Conflict of Interest

The authors declare that the research was conducted in the absence of any commercial or financial relationships that could be construed as a potential conflict of interest.
